# Amniotic Fluid Stem Cells with Low γ-Interferon Response Showed Behavioral Improvement in Parkinsonism Rat Model

**DOI:** 10.1371/journal.pone.0076118

**Published:** 2013-09-30

**Authors:** Yu-Jen Chang, Tsung-Yen Ho, Mei-Ling Wu, Shiaw-Min Hwang, Tzyy-Wen Chiou, Ming-Song Tsai

**Affiliations:** 1 Bioresource Collection and Research Center, Food Industry Research and Development Institute, Hsinchu, Taiwan; 2 Department of Life Science and the Institute of Biotechnology, National Dong Hwa University, Hualien, Taiwan; 3 Prenatal Diagnosis Center, Cathay General Hospital, Taipei, Taiwan; 4 School of Medicine, Fu Jen Catholic University, New Taipei City, Taiwan; 5 Department of Obstetrics and Gynecology, College of Medicine, Taipei Medical University, Taipei, Taiwan; Stem Cell Research Institute, Belgium

## Abstract

Amniotic fluid stem cells (AFSCs) are multipotent stem cells that may be used in transplantation medicine. In this study, AFSCs established from amniocentesis were characterized on the basis of surface marker expression and differentiation potential. To further investigate the properties of AFSCs for translational applications, we examined the cell surface expression of human leukocyte antigens (HLA) of these cells and estimated the therapeutic effect of AFSCs in parkinsonian rats. The expression profiles of HLA-II and transcription factors were compared between AFSCs and bone marrow-derived mesenchymal stem cells (BMMSCs) following treatment with γ-IFN. We found that stimulation of AFSCs with γ-IFN prompted only a slight increase in the expression of HLA-Ia and HLA-E, and the rare HLA-II expression could also be observed in most AFSCs samples. Consequently, the expression of CIITA and RFX5 was weakly induced by γ-IFN stimulation of AFSCs compared to that of BMMSCs. In the transplantation test, Sprague Dawley rats with 6-hydroxydopamine lesioning of the substantia nigra were used as a parkinsonian-animal model. Following the negative γ-IFN response AFSCs injection, apomorphine-induced rotation was reduced by 75% in AFSCs engrafted parkinsonian rats but was increased by 53% in the control group after 12-weeks post-transplantation. The implanted AFSCs were viable, and were able to migrate into the brain’s circuitry and express specific proteins of dopamine neurons, such as tyrosine hydroxylase and dopamine transporter. In conclusion, the relative insensitivity AFSCs to γ-IFN implies that AFSCs might have immune-tolerance in γ-IFN inflammatory conditions. Furthermore, the effective improvement of AFSCs transplantation for apomorphine-induced rotation paves the way for the clinical application in parkinsonian therapy.

## Introduction

Amniotic fluid cells obtained by amniocentesis have routinely been cultured and used for prenatal genetic testing. Multipotent stem cells can be isolated from many different tissues, and can provide a new cell source for regenerative medicine. Amniotic fluid-derived stem cells (AFSCs) represent ~1% in amniotic fluid cells recovered from second trimester amniocentesis samples, and the fetal origin of these cells has been confirmed by molecular evidence [1]. AFSCs express early embryonic markers that are absent in other adult stem cells, including Oct-4, Nanog and stage-specific embryonic antigen (SSEA-4) [2,3]. These findings suggest that AFSCs represent an early developmental stage that is intermediate to embryonic and adult stem cells in terms of their versatility and that they have unique potential for further applications [3,4]. AFSCs express the mesenchymal markers CD90, CD73 and CD105 and undergo various forms of differentiation *in vitro*, including mesengenesis and neurogenesis [2,4]. In an ovine autologous transplantation model, labeled AFSCs were injected into the peritoneal cavity of fetal sheep, and widespread AFSCs migration was detected within various organs after 2 weeks [5]. AFSCs have also been shown to stimulate the regeneration of mammary gland [6] and sciatic nerve [7] in animal models.

Parkinson’s disease (PD) is one of the most common neurodegenerative diseases today. It is caused by the progressive and selective loss of mesencephalic dopamine (DA) neurons of the nigrostriatal pathway. The etiology of PD remains unknown, although contributing genetic and environmental factors have been described [8]. PD affects approximately 1% of the population aged 65-69 and 1-3% of the population over 80 years of age. The impact of PD on the human health and the social healthcare system has grown rapidly as increasing life expectancy has become more common worldwide. PD is currently managed through medical and surgical therapies to control symptoms such as tremor, bradykinesia, rigidity and postural instability [9]. Medical treatment is effective but may lead to severe side effects, such as motor fluctuations or drug induced involuntary movements [10]. Surgical ablation of deep brain structures or deep brain stimulation can reduce neural activity, but the risks associated with surgical therapy preclude its use in more than a small proportion of patients [8]. Transplantation of human fetal ventral mesencephalic cells into the striatum has been reported to restore damaged neurons in PD patients [11,12]. However, invalidated control trials [13] and ethical concerns limit further application of this therapy. DA neuron replacement therapy may help to reduce the PD symptoms and provide a beneficial alternative for the corrective management of PD.

It has been known that the HLA expression in the central nervous system is very important for the neurodegenerative and inflammatory diseases [14,15]. Recent evidence has shown that γ-interferon (γ-IFN) is a key mediator in the death of dopaminergic neurons and up-regulates HLA proteins in PD [16,17]. AFSCs have been demonstrated to express specific proteins characteristic of neuronal stem cells, such as nestin and sox2, and to exhibit multiple phenotypes associated with neural-derived cells [2,18]. We have reported that clonally isolated AFSCs can be differentiated into neurons and induced into dopamine-producing cells [2]. In this study, we measured human leukocyte antigen (HLA) expression in of AFSCs after γ-IFN treatment. Furthermore, we transplanted AFSCs as therapeutic agents into 6-hydroxydopamine (6-OHDA)-lesioned rats. The effect of cell transplantation was evaluated by analyzing the recovery in rotation behavior and the expression of the specific DA proteins in the brain tissue.

## Materials and Methods

### Ethics statement

All human samples used in this study were obtained from the Cathay General Hospital, Taipei, Taiwan, ROC. All procedures were approved by the Institutional Review Board of the Cathay General Hospital, and all participants provided their written informed consent to participate in this study.

All animal experiments in this work were conducted under a protocol approved by the Tzu Chi University animal center and met Institutional Animal Care and Use Committee policies.

### Sample collection

All amniotic fluid donors were Taiwanese and their ages ranged from 25 to 35 years. Amniotic fluid sampling was performed for general diagnostic purposes between 16 to 18 weeks of gestation.

### AFSCs culture and characterization

AFSCs were established as previously described [19]. Adherent primary cells were expanded and passaged for subsequent experiments. All of AFSCs cell lines used in this study were polyclonal and heterogeneous.

Cells (at passage 4-7) were trypsinized and washed twice with phosphate buffered saline (PBS). To characterize cell surface markers expression, cells were labeled with the following fluorochrome-conjugated antibodies: CD14-fluorescein isothiocyanate (FITC), CD19-phycoerythrin (PE), CD26-FITC, CD29-PE, CD31-FITC, CD34-PE, CD44-FITC, CD45-FITC, CD73-PE, CD80-FITC, CD86-PE, CD90-PE, CD105-FITC, CD117-PE, CD119-PE, CD184-PE, HLA-Ia-PE, HLA-DR-PE (BD Bioscience, San Jose, CA), HLA-E-PE and HLA-G-PE (Biolegend, San Diego, CA). Samples were processed using a FACSCantoII flow cytometer (BD Biosciences), and at least 20,000 events were captured per sample. Data acquisition and analysis were performed using FACSDiva 6.0 (BD Biosciences) and FCS Express V3.00 (De Novo Software, Thornhill, Canada).

Assays to determine adipogenic and osteogenic potential were performed as previously described [20]. After three weeks of culture, osteogenic differentiation was assessed by Alizarin Red S (Sigma, St. Louis, MO) staining, and adipogenic differentiation was assessed by Oil Red O (Sigma) staining.

### γ-Interferon (γ-IFN) stimulation of human AFSCs and bone marrow mesenchymal stem cells (BMMSCs)

Human bone marrow mesenchymal stem cells (BMMSCs) were established and cultured as described in our previous study [20]. AFSCs and BMMSCs (at passage 4-5) were seeded as 5×10^3^/cm^2^ in culture vessels. Upon reaching 50% confluence, the cultures were washed with PBS and replenished with media containing 100 or 200 U/mL γ-IFN (R&D Systems, Minneapolis, MN). The cells were harvested for immunophenotype analysis or RNA extraction 48 hr later.

Though the AFSCs used in this study were heterogeneous, these cell lines showed similar response in expression in CD80, CD86, CD119, HLA-Ia, HLA-E and HLA-G after γ-IFN treatment. In HLA-DR expression, 11/15 AFSCs samples remained undetectable after γ-IFN stimulation, and only 4/15 AFSCs samples showed low expression by treating with γ-IFN (Table S1). To focus on the properties of these cells, the following PCR and animal tests were performed by the specific negative γ-IFN response AFSCs samples.

### Semi-quantitative polymerase chain reaction (PCR) and quantitative PCR (Q-PCR)

Total RNA was prepared using TRIzol Reagent (Invitrogen, Carlsbad, CA). First strand cDNA synthesis was performed according to the manufacturer’s protocol using M-MuLV Reverse Transcriptase (Thermo Scientific, Fremont, CA) and an oligo-dT primer. Semi-quantitative PCR parameters included 25 or 30 amplification cycles. PCR products were examined by 2% agarose gel electrophoresis and were visualized after ethidium bromide staining. Q-PCR was performed with SYBR Green PCR master mix (Thermo Scientific) using the ABI Prism 7700 Sequence Detection System (Applied Biosystems, Foster City, CA). The relative expression level of β-actin was used as an internal control to normalize specific gene expression in each sample. Relative quantification of marker genes was performed according to the △△Ct method. The primer pairs used in this study were listed as Table 1.

**Table 1:** Oligonucleotide sequences used in this study.

Primer	Sequence (5’ to 3’)	Accession No.
CIITA	Forward: GATGCGCTGAGTGAGAACAAGAT	NM_000246
	Reverse: TTGAGGGTTTCCAAGGACTTCA	
RFX5	Forward: CCCACTCAGCACAGCCAACT	NM_001025603
	Reverse: AGCCTTCGAGCTTTGATGTCA	
TGF-β1	Forward: CGCGTGCTAATGGTGGAAA	NM_000660
	Reverse: ATGCTGTGTGTACTCTGCTTGAACTT	
GAPDH	Forward: CAAGGTCATCCATGACAACTTTG	NM_002046
	Reverse: GTCCACCACCCTGTTGCTGTAG	
β-actin	Forward: TGTGGATCAGCAAGCAGGAGTA	NM_001101
	Reverse: CAAGAAAGGGTGTAACGCAACTAAG	

### Lesion and transplantation surgery

The Sprague Dawley rats (180~200 g at the beginning of the experiment, BioLASCO Taiwan Co., Ltd., Taiwan) were housed in a temperature- and humidity-controlled room with 12 hr light/dark cycles and were provided free access to food and water. The rats were subjected to 6-OHDA (Sigma) lesioning of the substantia nigra on the right side. Injection of 3 µL 6-OHDA (10 µg/µL in PBS containing 0.02% ascorbic acid) was made at the following two coordinates: P1: AP: -4.4 mm, ML: -1.2 mm, DV: -7.8 mm and P2: AP: -4.0 mm, ML: -0.8 mm, DV: 8.0 mm from bregma. The cannula was left in place for 10 min after infusion and then withdrawn to allow diffusion of the toxins.

After 4 weeks, the lesioned rats were intraperitoneally injected with 0.5 mg/kg apomorphine (Sigma) and tested for rotational behavior in an automated rotometer (TSE, Bad Homburg, Germany). Animals that exhibited 120-540 full turns contralateral to the lesion over 60 min were selected for this study.

Passage 6-8 AFSCs (2.5×10^5^ cells/2.5 µL PBS) were injected into the striatum at the following two coordinates (ipsilateral to the 6-OHDA lesion): P1: AP: 0 mm, ML: -3.0 mm, DV: -5.0 mm and P2: AP: 2.0 mm, ML: -3.0 mm, DV: 5.0 mm from bregma, whereas the PBS was injected into the same position in the sham control rats. Apomorphine-induced rotational tests were performed at 3, 6, 9 and 12 weeks after AFSCs transplantation. The rotational behaviors were recorded for 60 min and full 360° turns contralateral to the lesion were counted.

### Tissue processing

Rats were deeply anesthetized with 400 mg/kg chloral-hydrate (Sigma) and then perfused through the aorta with 200 mL PBS, followed by 200 mL 10% buffered neutral formalin solution. The brain was removed from the skull and fixed 10% buffered neutral formalin solution for 4 hr. Fixed samples were cryopreserved by repeated immersion in 10% and 30% sucrose and in PBS until the brains sank to the bottom of the container. The brains were then cut serially through the AFSCs transplantation site into 12-30 µm sections on a freezing cryostat (Leica CM3000, Leica, Solms, Germany).

### Immunohistochemistry and immunofluorescent staining

Endogenous peroxidase was quenched by pre-incubation of cryosections in 3% H_2_O_2_. Grafted brain sections were permeabilized in 0.3% Triton X-100 (Sigma) for 30 min and blocked with 5% bovine serum albumin for 1 hr. Sections were incubated overnight in primary antibody to tyrosine hydroxylase (TH; R&D Systems). After being washed with PBS, the sections were incubated for 1 hr in biotinylated anti-mouse secondary antibody (BioGenex, San Ramon, CA, USA). Streptavidin-horseradish peroxidase/DAB (BioGenex) was used to promote color development. The slides were then dehydrated, mounted and observed.

For immunofluorescent staining, grafted brain sections were rinsed in PBS containing 0.1% Tween 20 and permeabilized with 0.1% Triton X-100. Cells were blocked with 10% specific normal serum in PBS for 30 min prior to overnight incubation in the following primary antibodies: TH (Chemicon, Temecula, CA), dopamine active transporter (DAT, Chemicon), Glial fibrillary acidic protein (GFAP, Chemicon) and human mitochondria (R&D Systems). Cells were washed twice and then incubated with the appropriate rhodamine- or FITC-conjugated secondary antibody (Chemicon) for 1 hr at room temperature. Fluorescently labeled cells were visualized by fluorescence microscopy.

### Statistical analysis

The results were presented as the mean ± standard deviation (SD). Differences between experimental and control groups were compared using Student’s *t*-test. Differences were considered statistically significant at *p*<0.05.

## Results

### Characterization of human AFSCs

**Figure 1:**
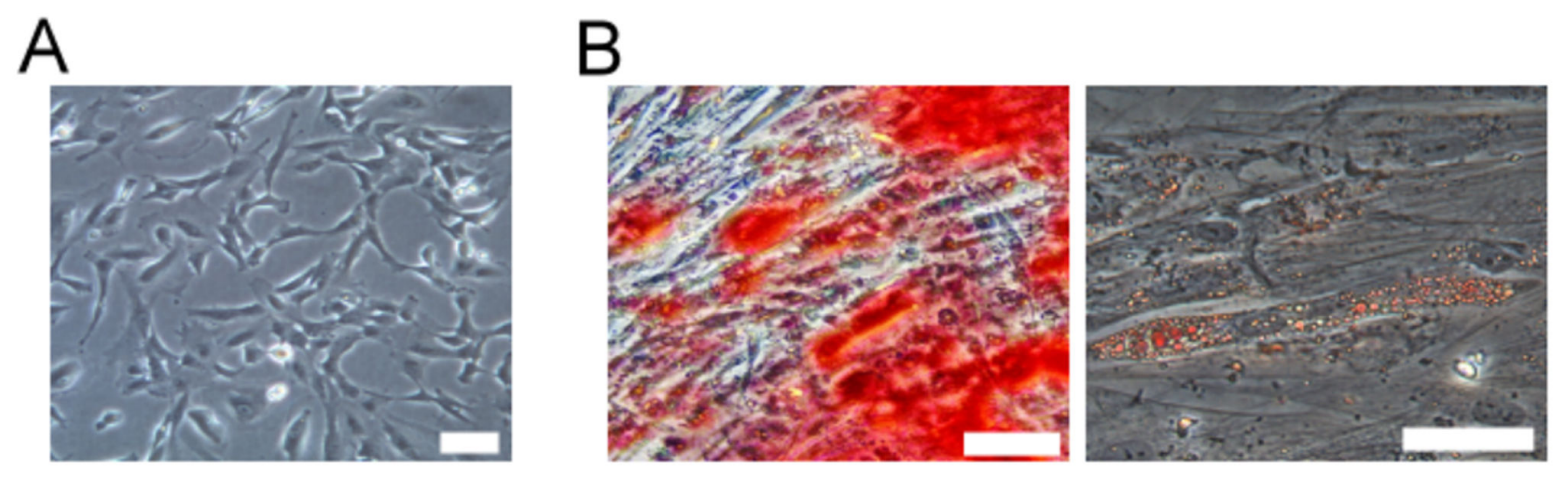
The morphology and mesenchymal differentiation potential of AFSCs. AFSCs are morphologically heterogeneous and are shown in small and polygonal shapes (A). After osteogenetic and adipogenetic induction for 21 days, the AFSCs were stained by Alizarin Red S (B, left) and Oil Red O (B, right), respectively. Bar: 10 µm.

AFSCs were successfully isolated and cultured from all tested amniotic fluid samples (n=15) and grew to confluence within 14 days (Figure 1A). AFSCs were cultured in osteogenic and adipogenic media to evaluate their mesenchymal differentiation potentials. Staining with Alizarin Red S revealed mineral nodules after 3 weeks of osteogenic induction (Figure 1B). Staining of AFSCs with Oil Red O revealed oil droplets when grown under adipogenic conditions (Figure 1B). The droplets were small, however, and only fewer than 10% of the cells stained positive after 3-weeks in adipogenic media.

AFSCs were immunophenotyped through the examination of cell surface markers by flow cytometry (Table 2). The AFSCs did not detectably express markers of leukocytes (CD14), lymphoid (CD19), T cell activation molecule (CD26), endothelial-lineages (CD31), myeloid (CD34 and CD45), antigen-presenting cells (CD80 and CD86) and stem cells factor receptor (CD117). The CD119 (γ-IFN receptor, 39.9 ± 6.8%) and CD184 (CXCR4, 17.9 ± 7.9%) were expressed by a fraction of the AFSCs population. Common mesenchymal stem markers such as CD29, CD44, CD73, CD90 and CD105 were expressed in the vast majority of AFSCs (>95%). We also examined the different HLA expression patterns***.*** The AFSCs expressed classic HLA-Ia (HLA-ABC) but not HLA-DR on the cell surface. Substantial variation between individual samples was evident in the expression of HLA-E (67.2±19.4%) and HLA-G *(*21±10.2%)***.***

**Table 2:** The surface markers expressed on AFSCs.

Marker	Positive cells (%)	Marker	Positive cells (%)
CD14	7.5 ± 1.8	CD86	5.9 ± 2.7
CD19	4.5 ± 3.8	CD90	99.5 ± 0.3
CD26	9.3 ± 5.1	CD105	97.2 ± 4.5
CD29	99.6 ± 0.2	CD117 (c-kit)	5.2 ± 2.7
CD31	2.3 ± 1.3	CD119 (r-IFNR)	39.9 ± 6.8
CD34	5.7 ± 3.6	CD184 (CXCR4)	17.9 ± 7.9
CD44	99.8 ± 0.2	HLA-Ia	99.7 ± 0.2
CD45	2.5 ± 0.8	HLA-DR	3.2 ± 1.3
CD73	99.9 ± 0.1	HLA-E	67.2 ± 19.4
CD80	5.3 ± 1.9	HLA-G	21.0 ± 10.2

### The effect of γ-IFN stimulation on HLA expression

**Figure 2:**
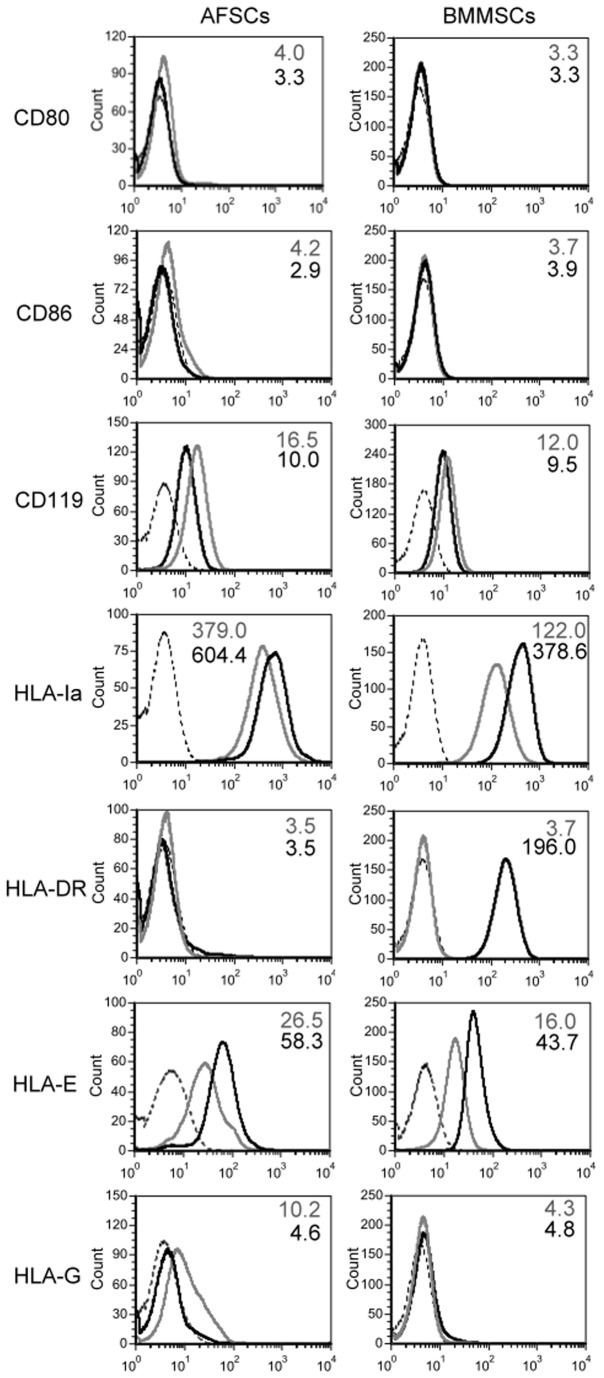
AFSCs surface marker expression after γ-IFN treatment. Surface marker expression was analyzed by flow-cytometry. Dotted lines represent the isotype control. Gray lines and black lines represent the expression of specific antigens without or with 200 U/mL γ-IFN treatment for 48 hr, respectively. Geometric means of fluorescence intensity are indicated in the top of each box (gray: no γ-IFN treatments; black: γ-IFN treatments).

To understand the effect of γ-IFN on HLA expression in stem cells, AFSCs and BMMSCs at passage 4-5 were stimulated with 200 U/mL γ-IFN for 48 hr. Neither cell type changed its morphology significantly during the 48 hr treatment (data not shown). Cell surface marker expression was subsequently analyzed by flow cytometry. As shown in Figure 2, expression of CD80 and CD86 was unchanged in AFSCs and BMMSCs after γ-IFN stimulation. As compared with untreated control, the expression of HLA-Ia and HLA-E increased in γ-IFN treated AFSCs and BMMSCs, but the fold increasing was lower in AFSCs than in BMMSCs (1.6 fold vs. 3.1 fold). Stimulation with γ-IFN had no obvious effect on the expression of either CD119 or HLA-G in BMMSCs, whereas their expression decreased slightly in γ-IFN treated AFSCs. HLA-DR was highly expressed in BMMSCs after γ-IFN treatment. In contrast, HLA-DR expression was unchanged in 11/15 AFSCs samples (Figure 2) and even after extending the γ-IFN exposure to 72 hr overall HLA-DR expression remained low (data not shown).

### The expression of regulatory factors associated with γ-IFN induced HLA-II expression in BMMSCs and AFSCs

**Figure 3:**
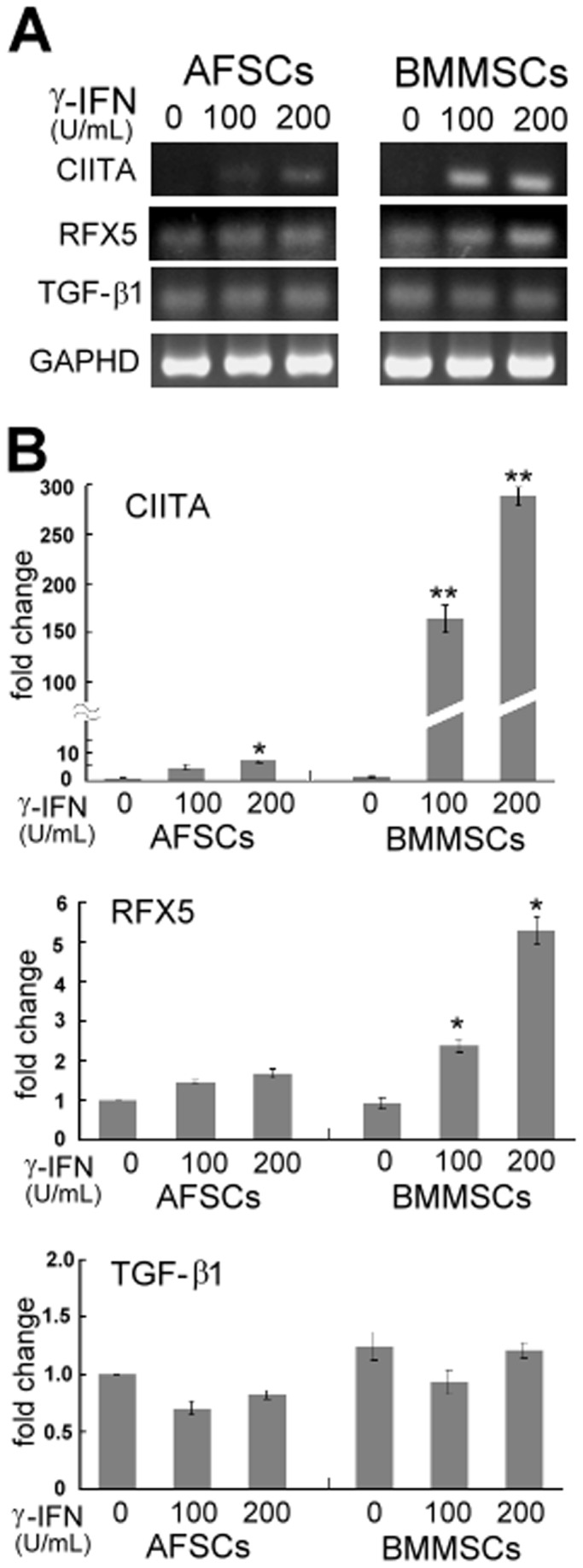
Expression of HLA-DR associated regulatory genes in BMMSCs and negative γ-IFN response human AFSCs after γ-IFN stimulation. (A) Semi-quantitative analysis of CIITA, RFX5 and TGF-β1 expression in AFSCs treated with different concentrations of γ-IFN. Amplification of CIITA, TGF-β1 and GAPDH was performed for 30 PCR cycles, and amplification of RFX5 was performed for 25 PCR cycles. GAPDH was used as internal control. (B) Q-PCR analysis of CIITA, RFX5 and TGF-β1 expression in AFSCs treated different concentrations of γ-IFN. The fold change in gene expression was determined for each gene by comparison of AFSCs with and without γ-IFN treatment. The mean ± SD of 3 replicates derived from at least 2 independent experiments is shown. * P<0.05, ** P<0.01 between each cell group without γ-IFN treatment.

To better understand the regulation of HLA-DR, the expression of major transcription factors in response to γ-IFN was evaluated by quantitative PCR. Expression of CIITA and RFX5, the key transactivators of the histocompatibility complex class II genes [21,22], were examined in BMMSCs and AFSCs. Expression of transforming growth factor (TGF-β1), which blocks γ-IFN-mediated HLA-II expression [23], was similarly examined in BMMSCs and AFSCs. Semi-quantitative analysis was initially performed by PCR with a low amplification cycle. As shown in Figure 3, neither cell type expressed CIITA mRNA before γ-IFN treatment. CIITA expression was strongly induced in BMMSCs when stimulated with γ-IFN for 48 hr. In contrast, CIITA was weakly induced in γ-IFN treated AFSCs and was only detected after 30 amplification cycles. Endogenous RFX5 and TGF-β1 transcripts were detected in both BMMSCs and AFSCs prior to γ-IFN stimulation. PCR analysis revealed that the expression of RFX5 in BMMSCs increased slightly after stimulation with γ-IFN, but no significant difference could be found in AFSCs. TGF-β1 mRNA expression was maintained at similar levels in both BMMSCs and AFSCs with or without γ-IFN stimulation.

CIITA, RFX5 and TGF-β1 expression was further analyzed by Q-PCR (Figure 3B). CIITA and RFX5 expression increased significantly in BMMSCs after stimulation with γ-IFN, whereas a much smaller change in the expression of CIITA and RFX5 was apparent in AFSCs. The expression of CIITA dramatically increased by 165 fold and 289 fold in BMMSCs stimulated with 100 and 200 U/mL γ-IFN, respectively. RFX5 expression was enhanced in BMMSCs by 2.3-fold (100 U/mL) and 5.2-fold (200 U/mL), but no significant increase was observed in AFSCs. Furthermore, there was a dose-dependent effect on the γ-IFN induced expression of CIITA and RFX5 in BMMSCs. TGF-β1 expression was unaffected by γ-IFN in both AFSCs and BMMSCs.

### Effect of AFSCs transplantation in a parkinsonian rat model

**Figure 4:**
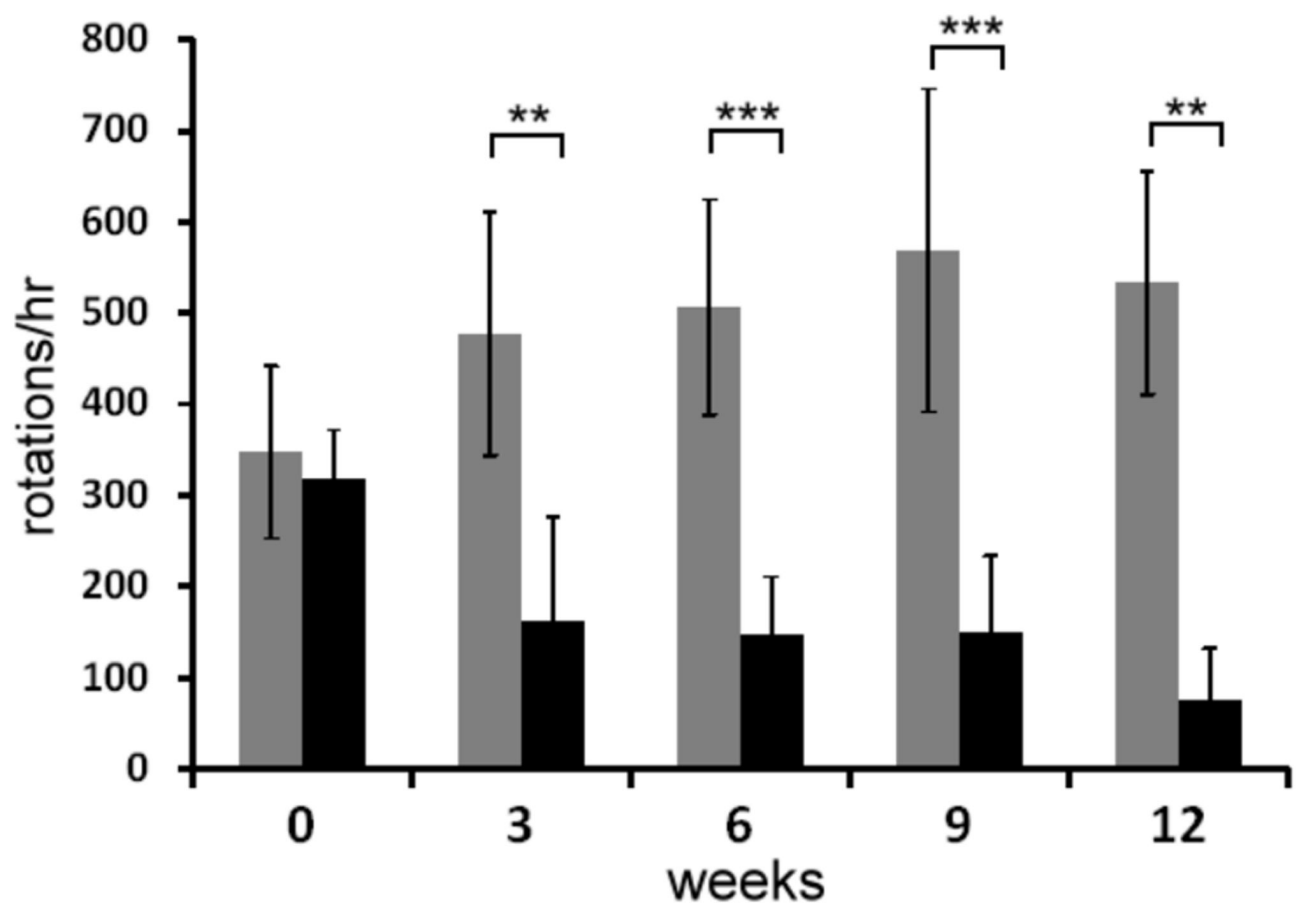
Rotation behavior in apomorphine-induced PD rats. A significant decrease in the number of apomorphine-induced rotations was observed in rats grafted with AFSCs (black bar) compared to the sham control group (gray bar) after 12 -weeks after transplantation. Data are expressed as the mean ± SD (n=5-8). Significant differences between the control and grafted groups are shown as * P<0.05, ** P<0.01 and *** P<0.001.

To determine the therapeutic potential of these negative γ-IFN response cells, the effect of AFSCs transplantation in 6-OHDA-lesioned animals was examined by quantification of rotations in response to apomorphine. Lesioned rats were divided into two groups of eight animals each. The rats in the sham control group received PBS into their dopamine-denervated striata whereas the rats in the experimental group received AFSCs in the same position. Rotation scores were examined at 0, 3, 6, 9 and 12 weeks after transplantation. One rat in each group was sacrificed 6 weeks after transplantation for tissue sectioning. Two rats in each group died at 9 and 12 weeks after transplantation. Before transplantation, apomorphine-induced rotations averaged 306±47 rotations per hour (Figure 4). The control group’s rotation score gradually increased over the duration of the study, reaching 533±47 rotations per hour in the final week of study. The rotation score for the AFSC transplantation group significantly improved. As shown in Figure 4, the average number of apomorphine-induced rotations was reduced to 76±55 rotations per hour 12 weeks after AFSCs injection. Statistically, at 12 weeks, the rotation score was reduced by 75% in the AFSCs grafted group but increased by 53% in the control group. In this study, the rotation scores for all of surviving AFSCs-implanted rats improved.

### Immunohistochemical staining in grafted striatum

**Figure 5:**
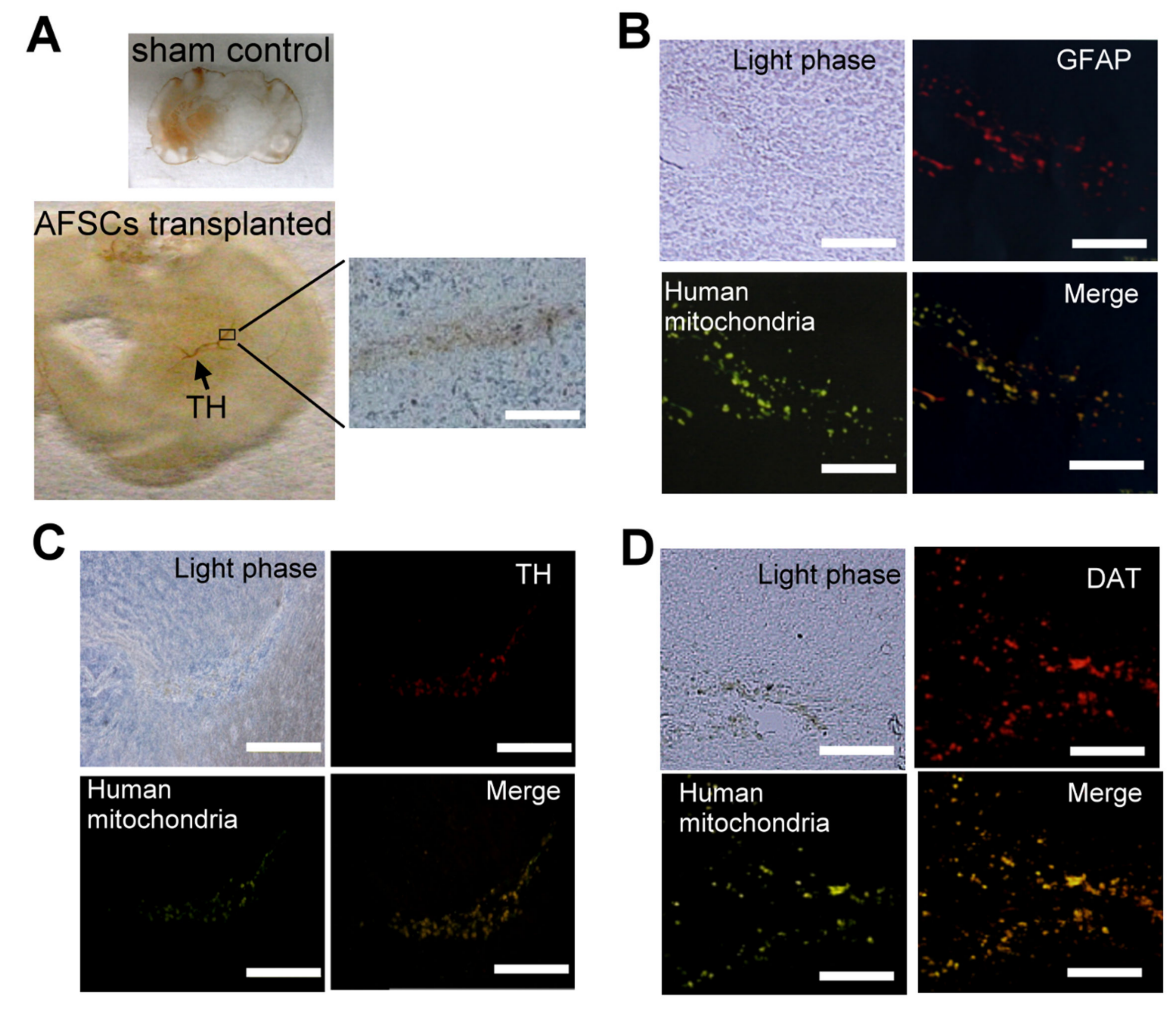
The therapeutic effect of AFSCs transplantation in parkinsonian rats. (A) TH staining in the parkinsonian rat’s brain 6 week after AFSCs transplantation. No staining was detected in the sham control. After AFSCs transplantation, positive TH staining was detected in the lesion and nearby area. Bar: 200 µm. (B) - (D) Immunostaining of neuronal markers in parkinsonian rat brain sections after transplantation with AFSCs. GFAP: Glial fibrillary acidic protein, TH: tyrosine hydroxylase, DAT: dopamine active transporter. Bar: 200 µm.

To assess the functionality of implanted AFSCs, grafted brain sections were obtained at 6 weeks after transplantation and immunohistochemical staining was performed. As shown in Figure 5A, TH loss was observed in right side of sham control rats with 6-OHDA-lesioned. In contrast, TH could be detected in lesioned and nearby areas in the damaged hemisphere of AFSCs-transplanted rats.

The distribution of grafted AFSCs was determined by staining for human mitochondria. Positive staining was observed around the lesion (Figure 5B-5D). Specific antibody staining was used to detect GFAP, TH and DAT. Merging these images with human mitochondrial staining revealed overlapping patterns of expression. These results indicate that the transplanted AFSCs survive for at least 6 weeks, and that these cells not only engrafted within the brain lesion site but differentiated into astrocytes and dopaminergic cells.

## Discussion

AFSCs have been demonstrated to share some characteristics with MSCs, including differentiation potential and immunophenotypic properties. In keeping with previous studies, the AFSCs in this report gave rise to osteoblasts and adipocytes. The AFSCs expressed some surface markers (CD29, CD44, CD73, CD90, and CD105) and failed to express other surface markers (CD14, CD19, CD26, CD31, CD34, CD45) previously associated with MSCs. Recently, the SDF-1/CXCR4 system was shown to play an important role in human fetal neural progenitor cell differentiation and bone marrow engraftment [24,25]. However, CXCR4+ cells were a rare (1%) subpopulation in BMMSCs [25]. We detected a high percentage of CXCR4+ cells (17.9%) in AFSCs samples, implying that AFSCs may have superior therapeutic potential compared to BMMSCs. Immunomagnetic sorting of AFSCs by CD117 (c-kit) produced a pluripotent population [3], although the CD117-positive AFSCs differentiated abnormally and encountered acute rejection after transplantation in rat myocardium [26]. Similar to previously reported finding, the AFSCs established in this study did not express CD117. Preparation of AFSCs without specific selection is thought to provide a good chance for homing and differentiation after transplantation [4].

MSCs have been shown to mediate immunosuppression in various therapeutic settings, including autoimmune diseases and allogenic transplantations [27]. However, MSCs may upregulate HLA-I and HLA-II molecules in response to proinflammatory cytokines such as γ-IFN [23]. BMMSCs may acquire MHC II-mediated antigen presenting capabilities upon stimulation with γ-IFN [28,29]. Because the BMMSCs and AFSCs in our study failed to express T-cell costimulatory factors (CD80 and CD86), both cell types may lack mature antigen presenting functions, even after γ-IFN stimulation. Both BMMSCs and AFSCs expressed similar levels of the γ-IFN receptor (CD119) (Figure 2) on their cell surface. However, our results showed that there were divergent responses to γ-IFN in the expression of HLA-I and HLA-DR between BMMSCs and AFSCs. It has been reported that MSCs do not express HLA-II regardless of their source (all negative). Induction of HLA-II expression by CIITA and RFX5 has also previously been demonstrated [22]. In agreement with previous studies, we found that expression of CIITA and RFX5 was induced by γ-IFN, resulting in the enhanced HLA-II in BMMSCs. Our result also showed that stimulation with γ-IFN did not significantly activate CIITA and RFX5 expression in AFSCs samples. As a result, HLA-DR kept negative expression after γ-IFN stimulation in these AFSCs. Negative regulation by TGF-β1 was not involved in the inhibition of HLA-II within either cell type in this study. We considered the possibility that AFSCs and BMMSCs may regulate HLA-II expression through disparate mechanisms. There have been diverse reports of γ-IFN inducing HLA-DR expression in cord MSCs [30,31], whereas undifferentiated and differentiated human embryonic stem cells failed to express HLA-DR after γ-IFN stimulation [32]. Our findings suggested that AFSCs are more similar to human embryonic stem cells than to cord MSCs and BMMSCs in terms of their response to γ-IFN stimulation. These findings also suggest that inflammatory thresholds may correlate with cellular development stages.

HLA-E and -G have previously been shown to inhibit natural killer cell-mediated cell lysis [33,34]. We found that AFSCs expressed more HLA-E and HLA-G than BMMSCs and that γ-IFN enhanced the expression of HLA-E in both cell types. CIITA and RFX5 have been demonstrated to upregulate HLA-Ia and HLA-E transcription [35,36]. The expression of HLA-Ia and HLA-E was higher in AFSCs than in BMMSCs under normal culture conditions, but induced expression in response to γ-IFN stimulation was more prominent in BMMSCs. We speculate that the expression profiles of HLA-Ia and HLA-E might result from higher expression of CIITA and RFX5 in BMMSCs than in AFSCs after γ-IFN treatment.

HLA-G, an immunoregulatory molecule involved in the protection of semi-allogenic embryonic tissue against the mother’s immune system, has previously been shown to occur only in its secreted form when expressed by BMMSCs [37]. Membrane bound HLA-G was abundant in first trimester tissues and diminished over the course of later developmental stages. In this study, we found that HLA-G was expressed to various degrees in AFSCs but not in BMMSCs. We hypothesize that the expression of HLA-G might be an indicator of the two cell types’ different developmental stages. Unlike most HLA-I genes, HLA-G has been shown to be regulated independently of the CIITA and RFX5 system [35]. In this study, the activation of CIITA and RFX5 by γ-IFN did not stimulate membrane-bound HLA-G expression. Instead, stimulation with γ-IFN reduced HLA-G expression to background levels. This result needs to be further studied.

Different cell types have been used to replace lost DA neurons in 6-OHDA–lesioned rats. It has been shown that transplanted embryonic stem cells [38] or neuronal stem cells (NSCs) [39] can grow into the host striatum, generate functional DA neurons and restore motor behavior(12). However, the risk of tumorigenesis, incomplete differentiation of embryonic cells, and difficulties inherent to the collection and proliferation of neuronal stem cells are major obstacles to the development of these strategies as effective therapies [8]. Alternative sources of stem cells need to be explored. The advantages of using fetal or adult stem cells in transplantation are the ability to select a safe cell population and obtain a sufficient amount of cells. To date, no evidence of *in vitro* or *in vivo* tumorigenesis has been associated with AFSCs. Furthermore, AFSCs can be easily isolated from amniocentesis samples without ethical concerns, and AFSCs exhibit higher proliferation rates than fetal and adult subcutaneous connective tissue [40]. These observations suggest that AFSCs are a promising cell source for therapeutic applications.

The therapeutic effect of BMMSCs and cord MSCs has been tested in parkinsonian models [41,42]. Transplantation of pre-differentiated human cord MSCs was shown to improve rotation behavior in PD rats, whereas undifferentiated cells did not improve rotation behavior [41]. However, the acquisition of adequate quantities of qualified differentiated neural cells for transplantation and the need to insure the *in vivo* survival of transplanted cells present major challenges for this cellular therapy. Mouse BMMSCs were shown to survive better in the 6-OHDA damaged hemisphere compared to the unlesioned side because the damage increased the viability of transplanted cells [42]. It has recently been demonstrated that human amniotic fluid cells express essential midbrain dopamine neuron survival factors, which are components required for dopamine synthesis and secretion and neurotransmitter exocytosis [43]. We also reported that AFSCs have the capacity to differentiate into multiple neural lineages and release dopamine *in vitro* [2]. However, AFSCs were recently reported to differentiate into immature dopamine neurons *in vivo* and failed to survive in PD rats [44]. Here, we have provided positive evidence indicating the therapeutic potential of AFSCs transplantation. In PD rats, apomorphine-induced rotations worsened in the control group, while a significant functional improvement was observed in the transplanted group. Correction of PD by cellular transplantation requires the functional integration of grafted cells within the brain’s circuitry. AFSCs have been shown to secrete neurotrophic factors that promote neuron survival and increase nerve regeneration *in vivo* [7]. In this report, the transplanted AFSCs migrated from the injection position, integrated into the brain’s circuitry and expressed specific dopaminergic neuron markers (TH and DAT). Engrafted cells were found not only in the lesioned regions but also in surrounding areas. We speculate that the damaged striatum might release factors that attract the transplanted cells to the nearby area. These results indicate that AFSCs may be a useful cell source for PD therapy.

The HLA expression of neuronal cells is related with various neurodegeneration diseases [14,15]. Also, the proinflammatory cytokines, such as γ-IFN, have been demonstrated to mediate HLA up-regulation of neuronal cells in central nervous system [45]. It has been reported that the γ-IFN participated in the death of dopaminergic neurons by regulating microglia activity and HLA proteins in PD [16,17]. In this study, we showed that stimulation of AFSCs with γ-IFN induces a weak expression of CIITA and RFX5 and consequently maintained the rare expression of HLA-DR. These findings imply that AFSCs may have immune-tolerance in γ-IFN-mediated inflammation. We also provided evidence that AFSCs implantation in a PD rat model resulted in a functional improvement as indicated by a reduced rotation score. The effective improvement in apomorphine-induced rotation by AFSCs transplantation paves the way for the clinical use of AFSCs in PD therapy.

## Supporting Information

Table S1 **The expression percentage of surface markers on AFSCs after γ-IFN treatment by flowcytometry.**
(DOCX)
