# Supplementary material for: Amniotic Fluid Stem Cells with Low γ-Interferon Response Showed Behavioral Improvement in Parkinsonism Rat Model
Source: PLoS One. 2013 Sep 30;8(9):e76118. doi: 10.1371/journal.pone.0076118 (PMC3786896; doi:10.1371/journal.pone.0076118)
Supplement: Table S1 — The expression percentage of surface markers on AFSCs after γ-IFN treatment by flowcytometry. (DOCX) [file pone.0076118.s001.docx]

Table S1. The expression percentage of surface markers on AFSCs after γ-IFN treatment by flowcytometry.

| AFSCs No. | Expression percentage (%) | | | | | | | | | | | | | | |
| --- | --- | --- | --- | --- | --- | --- | --- | --- | --- | --- | --- | --- | --- | --- | --- |
|  | Before γ-IFN treatment | | | | | | |  | After γ-IFN treatment* | | | | | | |
|  | CD80 | CD86 | CD119 | HLA-Ia | HLA-DR | HLA-E | HLA-G |  | CD80 | CD86 | CD119 | HLA-Ia | HLA-DR | HLA-E | HLA-G |
| AF021 | 2.4 | 1.5 | 50.2 | 99.6 | 2.1 | 95.2 | 19.8 |  | 1.9 | 1 | 42 | 99.5 | 2.3 | 99 | 6.6 |
| AF013 | 4.7 | 5.2 | 31.5 | 99.8 | 4.2 | 46.8 | 13.1 |  | 3.2 | 3.9 | 22.9 | 99.9 | 6.1 | 98.6 | 16.3 |
| AF119 | 7.3 | 4.3 | 40.2 | 99.9 | 1.8 | 57.9 | 14.9 |  | 3.2 | 1.4 | 36 | 97.6 | 2.6 | 99.2 | 7.7 |
| AF201 | 3.5 | 5.6 | 31.8 | 99.8 | 5.9 | 42.5 | 12.5 |  | 3.7 | 3.5 | 16.3 | 99.8 | 36.6 | 83.7 | 4.2 |
| AF125 | 5.9 | 7.6 | 38.1 | 99.3 | 1.8 | 61.5 | 14.2 |  | 3.3 | 1.5 | 18.5 | 99.7 | 3.8 | 92.3 | 6.2 |
| AF219 | 8.8 | 9.8 | 46.2 | 99.5 | 4.4 | 69.6 | 36.1 |  | 10.8 | 7 | 29.8 | 99.9 | 36.9 | 92.3 | 29.5 |
| AF204 | 5.9 | 3.8 | 36.2 | 99.5 | 2.5 | 89.6 | 13.3 |  | 3.2 | 1.8 | 24.3 | 98.9 | 3.3 | 98.8 | 9.1 |
| AF123 | 4.4 | 8.1 | 45.1 | 99.4 | 3.8 | 98.4 | 37.7 |  | 3.4 | 4.7 | 33 | 97.9 | 33.1 | 99.9 | 10.8 |
| AF191 | 5.1 | 9.2 |  | 99.9 | 4.8 | 59.8 | 27.5 |  | 5.5 | 5.2 | 33.8 | 99.9 | 10.1 | 88.3 | 6.1 |
| AF441 | 5.5 | 3.8 | - | 99.8 | 1.8 | 68.1 | - |  | 2.6 | 2.1 | - | 99.2 | 2.5 | 84.5 | - |
| AF124 | - | - | - | - | 4.2 | 49.5 | - |  | - | - | - | - | 6.8 | 90.5 | - |
| AF621 | - | - | - | - | 2.5 | - | - |  | - | - | - | - | 4.3 | 88.2 | - |
| AF442 | - | - | - | - | 2.9 | - | - |  | - | - | - | - | 6.3 | - | - |
| AF202 | - | - | - | - | 2.8 | - | - |  | - | - | - | - | 5.1 | - | - |
| AF093 | - | - | - | - | 2.5 | - | - |  | - | - | - | - | 4.2 | - | - |

*: The AFSCs were treated with 200 U/ml γ-IFN for 48 hr.

**Table legends:**

Table S1. The expression percentage of surface markers on AFSCs after γ-IFN treatment by flowcytometry.
